# First-line therapy with LPV/r vs NVP and 2 NRTIs in a developing country: W144 of a randomized trial

**DOI:** 10.1186/1471-2334-14-S2-O2

**Published:** 2014-05-23

**Authors:** Nathan Clumeck, Claude Mwamba, Kabamba Kabeya, Vincent Calvez, Serge Matanda, Dolorès Vaira, Gilles Peytavin, Coca Necsoi, Marc Delforge, David Kadiebwe, Chantal Milolo, Joe Ilunga, Liévin Kapend

**Affiliations:** 1 Saint Pierre University Hospital, Brussels, Belgium

## Aim

In resource-limited countries, NNRTI-based regimen may result in emergence of more HIV drug resistance because of a low genetic barrier. We compare the efficacy and tolerance of LPV/r and NVP-based regimens and 2 WHO nucleoside backbones in naive HIV infected patients (p.).

## Materials and methods

Naive p. from 5 clinics in Lubumbashi (Congo-DRC) were randomized to receive LPV/r versus NVP combined with TDF/FTC or ZDV/3TC. VL and CD4 were performed at baseline (BL) and every 24 weeks (W). The primary endpoint was the % of p. with therapeutic failure defined as clinical and virologic failures (VL>1000 c/ml)(missing data=failure), assessed at W48 and 96. We present here the results of 144 W of follow-up.

## Results

425 Black African p. (72% female; median (md) age 38 years, md CD4 165/µL; md VL 5.2 log c/ml) were randomized (216 in LPV/r, 209 in NVP). BL characteristics were comparable. In the ITT analysis, previous results showed no difference between LPV/r and NVP treatment arms at W96 except a higher proportion of virologic failure (VF) in p. on NVP-based regimens. W144 ITT analysis showed a significant difference on endpoints between LPV/r (94/216) and NVP (111/209)(p=0.0479) and persistence of a significant difference in VF rate (20/216 vs 37/209 for LPV/r and NVP, respectively)(p= 0.015). BL genotypes showed NNRTI mutations (mt) in 3/31 NVP-failing p. and no PI mt in LPV/r-failing patients. At time of failure, NNRTI mt were seen in 23/26 NVP-failing p. and 0/13 primary PI mt in LPV/r failing patients. NRTI mt were seen in 19/26 p. in NVP arm (including K65R in 7p. and M184V in 18p.) vs 3/13p. in LPV/r arm (M184V in 3p.).

Md CD4 change from BL was significant higher in LPV/r arm (251 cells/µL [interquartile range (IQR) 153;384]) compared with NVP arm (174 cells/µL [IQR 102-330])(p= 0.0093). Percentage of p. with adherence >95% was similar (73.6 vs 74.4 for LPV/r vs NVP).

## Conclusions

In a resource-limited setting after 144 weeks of follow-up NNRTI-NRTI first-line regimen is associated with more virologic failure, more drug resistance mutations and a lower immunologic response than a PI-based regimen.

